# The Influence of Teacher Discipline on Teaching Effect and Students’ Psychology in Universities and the Normative Suggestions for Discipline Behavior

**DOI:** 10.3389/fpsyg.2022.910764

**Published:** 2022-06-09

**Authors:** Zheming An

**Affiliations:** School of Law and Intellectual Property, Foshan University, Foshan, China

**Keywords:** teacher discipline, teaching effect, student psychology, normative suggestion, discipline behavior

## Abstract

In today’s educational environment, with the popularization of laws, more and more students pay attention to the maintenance of their own rights. However, due to the misinterpretation of punishment, it is very easy to mistake teacher punishment for “corporal punishment.” Therefore, it is particularly important to investigate the impact of teacher discipline on students. This paper first collects some knowledge related to the research based on the research results of scholars, and then makes a detailed analysis of this research from two aspects. It, respectively, introduces the influence of teacher discipline on teaching effect and students’ psychology in universities, and the normative suggestions for discipline behavior in this paper. It then uses formulas to explain how the teaching and learning optimization algorithm works. Finally, it analyzes the changes among teachers’ discipline, students’ psychology, and coping style through experiments. The results showed that urban students had the highest probability of being disciplined for being late, at 53%, and the lowest probability of being disciplined for not completing homework, at 34%.

## Introduction

Punishment, literally understood, is to achieve the purpose of “discipline” by means of “punishment.” It is the right and duty of teachers to discipline students as professional educators. However, due to the lack of laws and regulations, the current chaos in teacher punishment is a serious problem. This not only makes school education in a dilemma, but also makes it difficult for teachers to exercise their rights. This embarrassing situation makes the legislation of teachers’ disciplinary rights show an “urgent” trend. On the one hand, with the rise of humanism, the improvement of democracy, and the rule of law, the people-oriented educational thought has been expanded arbitrarily. Especially in recent years, school education has become more supportive of appreciation education. Punishment is generally regarded as having too many negative effects on the physical and mental health of students, and it is a violation of the educational philosophy of democracy and equality. The impact of these two educational concepts eventually led to a “chaotic state” when teachers exercised discipline. So that teachers who lack the correct awareness of punishment categorically deny the value and significance of educational punishment. Not only the school keeps a distance from education and punishment, but teachers are more “safe” and indifferent to education and punishment, and they are indifferent to or even sit idly by when it comes to students’ violations. On the other hand, the excessive exercise of teachers’ disciplinary power has resulted in the problem of excessive teachers’ disciplinary power and abuse of disciplinary power. Excessive discipline is a means of discipline that causes physical and mental harm to students, with strong personal emotions and factors, such as suspension and dismissal for students with poor grades. Abuse of discipline is also a phenomenon of excessive discipline. It is the teacher’s “stubborn” education method of abandoning the positive function, and the negative education method is used to the students. In a word, the problem of teacher punishment in China is chaotic at present, and it is imperative to regulate teacher punishment.

Because of this, the research on punishment will have a clearer understanding of the social situation and play a very important guiding significance. Therefore, this paper makes an in-depth study on the influence of teacher discipline on teaching effect and students’ psychology in colleges. At the same time, according to the conclusions of the research, some reasonable and standardized suggestions for disciplinary behavior are put forward, so as to improve the teaching efficiency and improve the teaching effect, and avoid irreversible effects on the students’ psychology.

The innovation of this paper are that:

1. It gives detailed suggestions on disciplinary behavior from four levels: legislation, society, schools, and teachers. In addition, for the role of the teacher, three valuable suggestions were put forward: clarifying the purpose of punishment, strictly abiding by the principles of punishment, and mastering the skills of punishment. It provides a clear direction for teachers’ future disciplinary behavior.
2. The teaching and learning optimization algorithm is adopted to test the influence of disciplinary behavior on the teaching effect, and a large number of disciplinary behaviors have an explanation on the psychological impact of students.
3. The reason why punishment as an educational method has caused many debates, and the reason why China’s teachers’ right to discipline has not been clearly recognized by the law is that the research on the function of punishment in China has not been in-depth. This paper has made an in-depth study on the application of teachers’ disciplinary rights in the teaching effect of college students.

## Related Work

Many scholars at home and abroad have provided a lot of references for the research on teacher discipline, teaching effect, student psychology, and normative suggestions.

Silva et al. (2017) validates public school teachers’ concepts of disciplinary violations and investigates behaviors and/or incidents that occur in the classroom that are considered disciplinary violations, possible causes, and ways to deal with problems.

Vlasenko et al. (2019) elaborates on the problem of teaching mathematics as an organizational process for subject training. Vlasenko et al. (2019) analyzes historical principles and trends in the creation of specialized schools of the subject, as well as school practices in Russia and the Soviet Union. Vlasenko et al. (2019) investigates the experiences of France, Japan, the United States, and other countries in the differentiation process of high school education. Vlasenko et al. (2019) expounded the characteristics of mathematics education content in different mathematics training directions in subject professional schools. He accordingly thought deeply about the continuation of comprehensive secondary education and higher education process.

Muh and Munawar (2020) aims to analyze the impact of organizational culture and work motivation on the disciplined work of SMK Mitra Karya Karawang. His results show that organizational culture and work motivation together have a significant impact on disciplinary work. Some organizational culture and work motivation have a significant positive impact on work discipline.

Mulani (2019) analyzes the relationship between transformational leadership and teacher work discipline and educational service quality in East Jakarta high school principals. The results show that there is a positive relationship between the transformational leadership of principals and teachers’ work discipline and the quality of educational services in East Jakarta high schools. This finding suggests that, in order to improve the quality of teacher services, it is recommended to increase the value of transformational leadership and improve teacher work discipline to become a culture of teacher virtues that provide good educational services.

Sadovenko and Szpak (2021) raises the question of the preparation of teaching by specialists who are not educated at the Ukrainian Technical Institute. The development of primary science and diagnostics in applying differentiated psychology and instructional influence mechanisms in technical colleges will help align scientific and methodological work in groups and individuals. This works with junior teachers and staff as a whole, planning further stages.

Dzhumagulova (2021) examines the question of the potential ability of certain disciplines to shape the professional ability of teachers in the future society. The goal set by Dzhumagulova (2021) is to consider an important part of the formation system of teachers’ professional competence in the future society. That is: the organizational form of classroom work, teaching methods, and other means of forming a system for teachers’ professional ability in the future society.

Farmanova (2021) determines the impact of quarantine on teacher jobs in higher education institutions. He sheds light on the level of development, challenges, and characteristics of improving the digital capabilities of teachers in humanitarian disciplines, as well as teachers’ needs in education and makes recommendations on how to arrange education and make administrative decisions.

The data from these studies are not comprehensive, and the results of the studies are open to question. Therefore, it cannot be recognized by the public, and thus cannot be popularized and applied.

## The Influence of Teacher Discipline on Teaching Effect and Students’ Psychology in Universities and the Normative Suggestions for Discipline Behavior

### Influence of Teacher Discipline on Teaching Effect and Students’ Psychology in Universities

Punishment has two meanings. The first is “punishment,” that is, punishment; then there is “punishment,” that is, abstinence. This paper argues that in educational practice, disciplinary action is a negative sanction used to make students realize their mistakes and sincerely repent. It has the following characteristics:

1. It must have an educational purpose. Although punishment is also sanctioning, it is only a means, not an end. The ultimate purpose of discipline is to educate students to correct their mistakes sincerely, convincingly, and actively.
2. It must cause the student to feel some degree of pain or shame.
3. It must be the consequence of the student’s mistake.

Punishment is an educational method that cannot be ignored in the educational process. The purpose of discipline is to make students recognize their faults and correct them. This is a kind of sanction with great “educational” value, and this educational nature runs through the whole disciplinary activity (Lindsay and Hart, 2017; Goos and Bennison, 2018). In short, the essence of punishment is not to punish people, but to correct bad behaviors (Silva-Fletcher and May, 2017).

**Figure 1:**
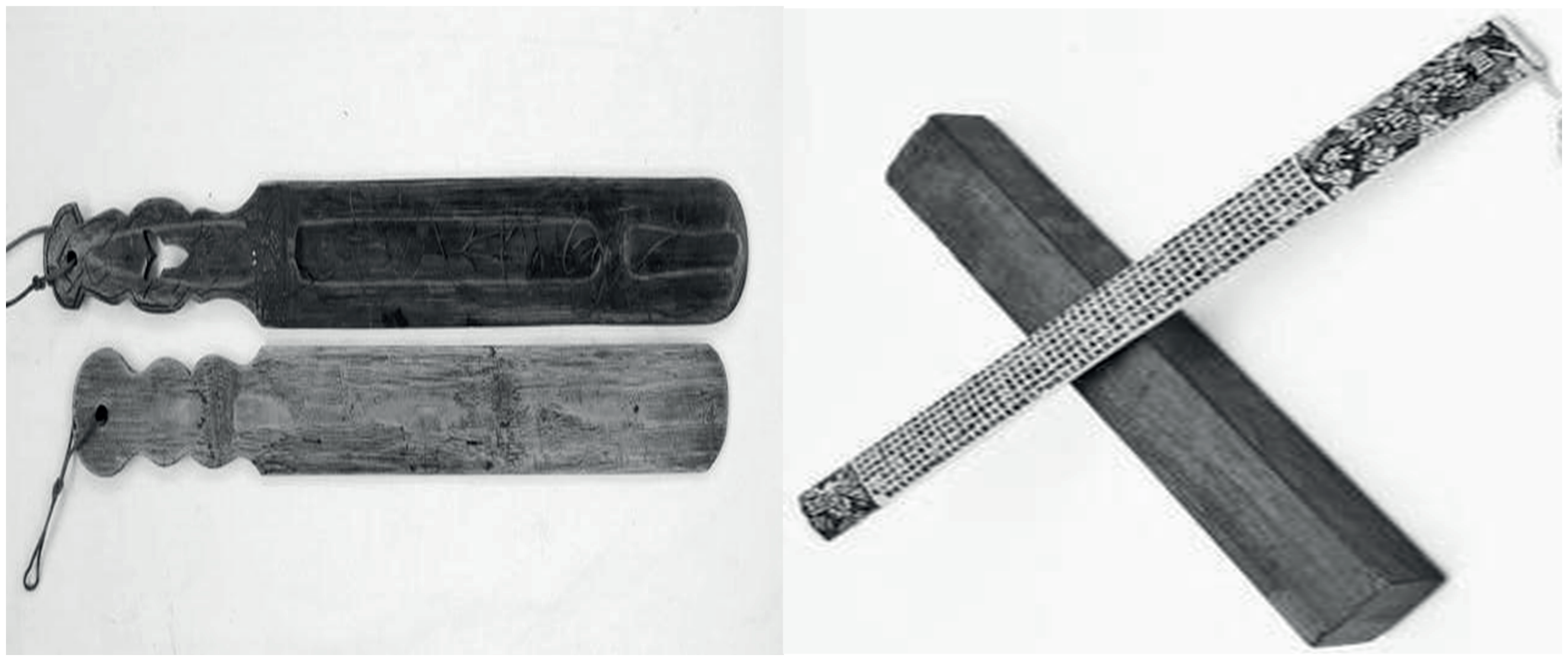
Example of a ruler.

**Figure 2:**
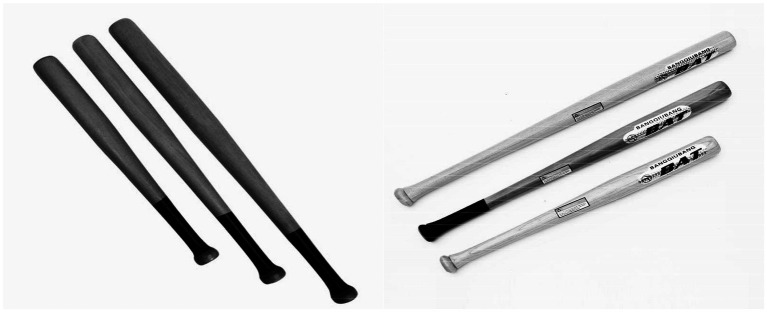
Example of a stick.

Traditional Chinese education often uses the method of “beating” to educate students who have anomie behavior. One of the more classic punishment tools is the “rule ruler” (as shown in Figure 1). In the medieval era of the West, “stick education” has become the main form of school education. The club was an indispensable tool in the medieval school (as shown in Figure 2; Duarte and Brewer, 2019; Afandi et al., 2021).

The teacher’s right to discipline is the behavior within the scope of the teacher’s responsibility, which is legal and has no right to be interfered with. Education, as a special activity, has inherent stipulations in the disciplinary behavior of teachers due to the characteristics of its own educational activities. It should defend teachers’ right to discipline through legislation to ensure the smooth progress of educational activities (Pereira and Ravasio, 2021).

**Figure 3:**
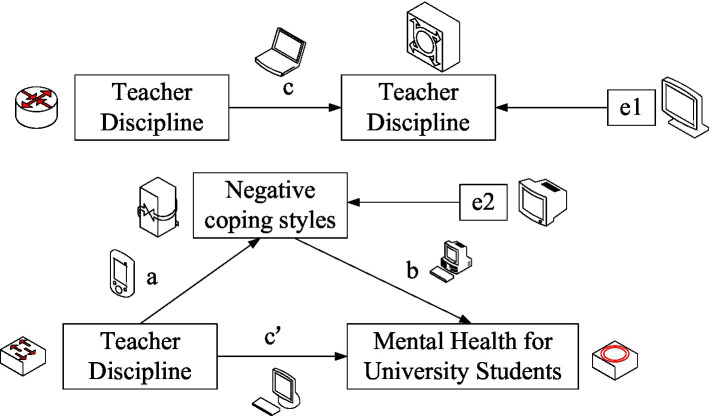
Pathways mediated by negative coping styles between teacher punishment and student mental health.

The model used in this paper is shown in Figure 3.

Corporal punishment is a physical and psychological abuse of students, and at the same time, it does not exclude the insult to their mind and personality. There is an essential difference between corporal punishment and punishment (Syafriadi et al., 2021; Jain et al., 2022). First of all, discipline emphasizes the word “precept,” which is aimed at guiding and educating, and does not have the purpose of harming the physical and mental health of students. Corporal punishment implies illegal behavior. In order to achieve the purpose of punishing students, it damages the physical and mental health of students and has the potential of breaking the law. Secondly, the purpose of discipline is to encourage students to recognize mistakes through introspection, focusing on moral modeling. Corporal punishment focuses on making students stop making mistakes because of fear of pain, showing a short-term oppressive control effect (Williams et al., 2020). Finally, the principle of discipline is not to damage the physical and mental health of students. The principle of corporal punishment is to make students feel fear or pain (Dng et al., 2020; Solomakha, 2020). Punishment emphasizes students’ self-examination and repentance, avoids causing students’ psychological problems, and mostly has a positive impact on students. Corporal punishment is an educational form of outward behavior. The effect of internal education is not obvious, and it is easy to cause students’ sense of resistance and disgust, which is a potential obstacle to education and teaching (Morales et al., 2020). In short, although punishment and corporal punishment have a certain degree of identity in form, they are not homogeneous.

Punishment can enable students to experience setbacks and failures, enhance their psychological endurance, and hone their will (Aksoy, 2020; Jain et al., 2020). The road of growth in life is not smooth, there are always setbacks and failures. Schools should teach students not only the ability to succeed, but also the courage to face failure. However, modern education emphasizes appreciation education and incentive education. There was a round of applause in the class and a smile in the class. Students live with praise and never know how to deal with blows and failures (Saragih and Mardianto, 2019).

Such education is not a healthy and comprehensive education. A complete education should not only have “appreciation” without “discipline.” The experience of setbacks and failures is also a part of life and enhances psychological endurance. Hone the will is the necessary content of education (Novitasari et al., 2021; Santiago-Rosario et al., 2021). Therefore, when students are punished, let them understand the truth of “how to see a rainbow without experiencing wind and rain.” Only by establishing a healthy mentality can they truly have the last laugh in the future.

**Figure 4:**
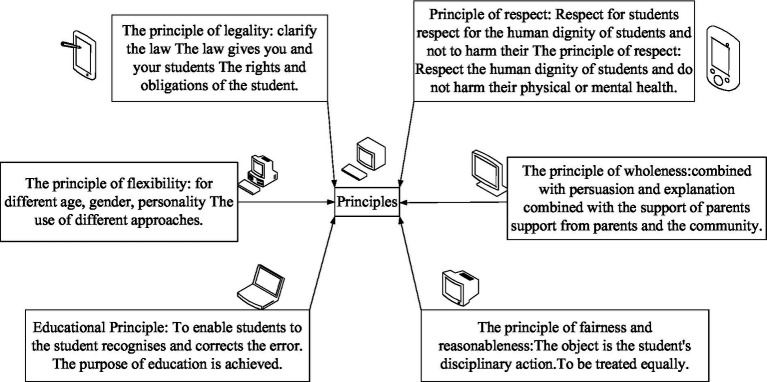
Principles for the exercise of teachers’ disciplinary powers.

There are six main principles: the principle of fairness and reasonableness, the principle of integrity, the principle of respect, the principle of education, the principle of flexibility, and the principle of legitimacy. The principle of teachers’ exercise of disciplinary power is shown in Figure 4.

### Normative Suggestions for Disciplinary Behavior

In the process of teachers exercising disciplinary power, there are many problems, and they are multi-faceted. If it wants to change the status quo and make the disciplinary power more effective and reasonable, the state, teachers, and schools need to form a joint force. Effective measures need to be taken together, and the actual implementation is to strengthen legislation, correctly guide public opinion, improve the overall quality of teachers, and strengthen school management.

#### From the Perspective of Legislation: Strengthening Legislation

For teachers’ disciplinary rights, the first and foremost is to seek institutional guarantees. However, China’s current laws and regulations do not make clear and specific provisions on teachers’ disciplinary rights. The principles, scope, standards, methods, and procedures of its exercise are not very clear. As for the legal supervision system and the relief system, it is rarely involved. In order to reduce the negative impact of disciplinary action and provide assurance and guidance for teachers to exercise their disciplinary power boldly and correctly, China must strengthen legislation and formulate corresponding laws, regulations, and policies.

##### Legislation on Teachers’ Disciplinary Rights

We can legislate the teacher’s disciplinary power and clarify its concept. It also specifically stipulates the circumstances under which teachers can implement discipline, as well as the basic principles and methods of implementing discipline and other related issues. At the same time, through legislation to limit the abuse of teachers’ disciplinary power. In this way, teachers can understand what the teacher’s disciplinary power is, under what circumstances, within what scope, and in what way.

##### Formulating Implementation Rules

Legislation can only make a principled regulation on teachers’ disciplinary rights. But in practice, there will be many specific problems and I do not know how to apply the law to solve them. Therefore, it is very necessary to formulate a detailed implementation rule for teachers’ disciplinary rights. The role of the implementing rules is to concretize the law and explain it in detail or make supplements. It should be practical and easy to operate to better guide practice. This article believes that the implementation rules mainly include the following two parts:

1. Criteria for exercising disciplinary power.

That is, what kind of punishment is used under what circumstances. The student’s non-standard behavior is roughly graded according to the degree of violation or the degree of adverse impact. Then, the punishment methods are roughly graded according to different degrees of severity, and finally, the two are corresponding. It determines which level of punishment should be imposed for a certain level of non-standard behavior.

2. Procedure for exercising disciplinary power.

When determining the procedure, it can be determined which level of punishment can be made by the teacher alone and on the spot according to the standards for the exercise of the disciplinary power. Which level of discipline needs to be notified to parents, reported to the dean of education or the principal, and made after collective discussion and investigation. And whether punishment needs to be announced, filed, etc.

##### Strengthening of the Supervision and Legislation of Teachers’ Disciplinary Rights

Without the power to supervise and check, it will inevitably lead to abuse. Therefore, the teacher’s disciplinary power must be restricted and necessary supervision should be given to ensure its proper and fair exercise. At present, since China’s teacher disciplinary power itself has not been expressly stipulated by law, there is no need to supervise its exercise. We must strengthen supervision and legislation and realize the legalization of supervision. Supervision and legislation are the premise of supervision in accordance with the law, and it is to “prevent trouble before it happens.” Only the establishment of sound supervision laws and regulations can provide a criterion for effectively supervising the exercise of teachers’ disciplinary power.

##### It Is Necessary to Improve the Legal Remedy System

1. The student appeal system needs to be improved. Legislation stipulates the conditions, scope, and time limit for students to exercise the right to appeal, as well as the acceptance agency and handling procedures of the appeal. It makes the student’s right to appeal truly implemented and guaranteed. In addition, a reconsideration can be filed for dissatisfaction with the outcome of the appeal. It is necessary to clarify the scope of reconsideration, the time limit, the acceptance authority, and the handling procedures from the legislation, and improve the relief channels after the appeal.
2. It is necessary to clarify the issue of case acceptance in lawsuits for infringement of teachers’ improper punishment. It prevents students from being dismissed by the court for being “out of scope” when they sue. Because the teacher’s disciplinary power has the dual attributes of right and power. This paper argues that in such cases students can file an administrative lawsuit (with civil damages attached).

#### From a Social Perspective: Correct Public Opinion Guidance

##### Rational Guidance for News Media

A good social atmosphere and public opinion atmosphere can ensure that teachers can effectively exercise their disciplinary rights, so the government should actively strive to create a good atmosphere. On the one hand, we must vigorously publicize laws and regulations, especially education laws and regulations. It can use the media to carry out various forms of activities, so that the correct education concept and punishment concept can gradually penetrate into the hearts of the public. On the other hand, the media are required to follow fair and objective standards when reporting cases of teacher discipline. It must evaluate the event itself, and it is strictly forbidden to distort the report in order to increase the news highlights; otherwise, it will bear adverse consequences.

##### Parents Should Change Their One-Sided Understanding of Punishment

Parents should strengthen their self-cultivation, conduct self-learning through various channels, and improve their personal cognitive ability and level. They should correctly evaluate and view teachers’ right to discipline from the perspective of children’s development, and realize that the purpose of discipline is education. This is the most crucial point. They must change two existing misconceptions. One is that it is natural for teachers to punish students, so they acquiesce or tolerate teachers’ improper disciplinary behavior. The second is to think that as long as a teacher uses punishment, it is corporal punishment, and thus accuse him. Parents should really care about their children’s education. They need to communicate with teachers frequently. On the one hand, they need to understand the child’s growth in school, and on the other hand, they should inform the teacher about the child’s ideological status, learning, and living conditions at home, so that the teacher can better educate them. It is especially important to note that when a child is disciplined in school, the reason should be promptly asked, and the child should be guided to view the matter in a correct way to achieve educational purposes. If teachers improperly exercise their disciplinary powers and violate the legitimate rights and interests of children, parents should know how to seek remedies. This requires regular strengthening of the study of legal knowledge.

#### From the Perspective of Teachers: Changing Concepts and Improving Their Own Quality

##### Teachers Should Change Their Concepts and Establish a New Concept of Teachers and Students

The traditional teacher–student view holds that teachers and students are in a relationship of teaching and learning. Students must obey the teacher absolutely, and the status of the two sides is unequal. Correspondingly, in the teaching process, the teacher’s teaching is the center and does not pay attention to how the students learn. Teachers only pay attention to the teaching of knowledge and do not pay attention to the cultivation of other non-intellectual aspects of students. The teaching method is rigid and rigid, stressing “indoctrination” teaching. Therefore, in order to regulate teachers’ disciplinary rights, teachers must change their concepts and establish a new concept of teachers and students.

Teachers should recognize the essence of education and start from the characteristics of students’ physical and mental development. Teachers need to change the previous “teacher-centered” indoctrination teaching model, learn to respect students, treat students equally, truly realize “student-oriented,” and emphasize the combination of teaching and learning. Only when this idea is established in the mind can teachers consciously proceed from the aspects of the students’ personality and the nature of their mistakes when implementing punishment. They will consider the acceptability of discipline and explore the most suitable discipline to achieve the best educational effect.

##### Strengthening Learning and Improve Self-Literacy

First of all, teachers should strengthen their own moral cultivation. Teachers should not only have extensive knowledge and strong teaching ability, but also have a certain level of morality. The so-called words are not as good as deeds, and teachers must become moral role models for students. Only when they are “right” can they “do not order.” What teachers demand of students should be what they should demand of themselves. They do it well themselves first, and they can be confident when educating students. Second, they need to improve their professionalism. Knowledge of pedagogy and educational psychology can help teachers better grasp the laws of education and the characteristics of students’ physical and mental development. Knowledge, such as educational management, can help teachers improve their management skills. Through the study of this knowledge, teachers can be more powerful in educational practice, target different situations, and exercise disciplinary power more effectively. Teachers must truly recognize this, take the initiative to conduct professional learning, and apply it in practice. Third, teachers should improve their legal literacy. Teachers must know the law and understand the law, and be clear about their own rights and responsibilities and the legal rights of students. Only by establishing legal awareness can they ensure that their behavior does not violate the requirements of the law and the rights of students in practice. Finally, teachers should strengthen their own psychological quality. In the process of education, teachers are bound to encounter many difficulties. If the psychological quality of teachers is not good, it is easy to have bad emotions. This adversely affects the educational work, and even causes the educational work to be unsuccessful. Strong willpower, stable emotions, and good character all help teachers exercise the right of punishment correctly and obtain educational results.

#### From the School Level: Strengthening School Management

##### Improvement of Relevant Rules and Regulations

1. The formulation of reasonable school rules. School regulations must take into account the interests of both the educator and the educated party, and achieve a balance between the two. It cannot limit or infringe on the legal rights of students for the realization of the school’s interests and values. The content of the school rules should be specific and the terms should be clear. It can act as a guide, telling students what behaviors are allowed and what are prohibited. The implementation of school rules must follow the necessary procedures and must not violate the legal rights of anyone. In the event of infringement, there must be a corresponding remedy mechanism.
2. It is necessary to improve the teacher evaluation system and change the previous practice of judging teachers only by their teaching performance. It is necessary to establish a diversified evaluation standard system. In the process of improving the assessment system, the standard should not be single. For example, teaching performance alone is not enough to make a comprehensive and correct evaluation of teachers, but also conduct evaluation. Teachers who hold the idea of non-punishment should be educated to improve their ideological awareness.

##### Strengthening the Management of Teachers

First of all, it is necessary to ensure the basic quality of teachers. Secondly, it is necessary to pay attention to the construction of teachers’ morality. Schools should carry out practical and effective teacher morality building activities based on the actual situation of the school. It is necessary to improve the evaluation standards of teachers’ morality and cultivate teachers’ civilized behavior and awareness of caring and respecting students.

##### It Is Necessary to Establish an Internal Supervision Institution and a Complaint Mechanism for Disciplinary Rights

Schools can work with parents to elect representatives through self-recommendation and democratic elections, establish a disciplinary supervision committee, and designate supervisors. Supervisors are responsible to the committee, and the committee is responsible to all students and their parents, supervising and inspecting teachers’ exercise of disciplinary power. Schools can also invite parents and social figures to discuss how to establish a grievance mechanism for disciplinary rights on the basis of democratic discussions. The conditions, scope, procedures, etc. of the appeal have been determined to make it operational.

Teachers are the first responsible person for education, the executors of relevant systems, and the authors who are in direct contact with students on the front line. The teacher’s words and deeds are closely related to the students and may affect the students’ life. Therefore, teachers should be especially careful to use disciplinary methods to educate students. In particular, they need a scientific understanding of the disciplinary law and the power of punishment, so as to correctly guide their behavior.

- The purpose of punishment must be clearly defined.

Disciplining students is different from legal sanctions. It is not simply to punish students for some misconduct, but to touch students’ minds and bodies. Therefore, discipline students should meet the following objectives:

1. It can improve students’ self-reflection ability.
2. It can cultivate students’ sound personality.
3. It can cultivate students’ good study and living habits and form behaviors that conform to social norms.
4. It can ensure the normal conduct of educational and teaching activities.

It should be noted that the purpose of discipline is to educate students, not discipline itself. The method of punishment should not be the first choice for correcting students’ anomie behavior, but should be the choice made after persuading education, canceling positive reinforcement and other methods are ineffective. Frequently giving aversive stimuli will not achieve educational effect. Some teachers try to establish their own prestige through discipline will not work. Teachers should pay attention to the improvement of their own quality, which includes not only a professional quality of teaching, but also moral quality. Only teachers who have extensive knowledge and noble character, and at the same time truly care for their students, can establish prestige among the students and truly achieve the “dignity of teachers.”

B. The principle of punishment must be strictly observed.

The counterpart of the teacher’s disciplinary power is the student. Teachers should fully consider the developmental characteristics of students when exercising their disciplinary powers, and keep in mind their responsibilities for educating people. Discipline is necessary, but it must reflect the educational nature of discipline, so that students can be touched and improved. Teachers must abide by the following principles when exercising disciplinary powers:

1. Principles of disciplinary education.

Education is the first principle in the implementation of disciplinary law. Teachers must not punish for the sake of punishment, they must be educational, show love and patience. Students are more curious, but tend to have weaker self-control, in which case mistakes can occur. Teachers should protect students’ curiosity and curiosity, and choose disciplinary methods carefully. Students’ enthusiasm for knowledge must not be undermined by teachers’ appropriate discipline. They must carefully analyze the motivation of students and make the best use of the situation, so as to achieve good educational results. Especially after some students make mistakes, they already understand their own faults. When they already feel self-blame, or when good substitute behaviors have appeared, as long as the purpose of education is achieved, teachers should not discipline students any more.

2. The principle of punishment.

Before punishing a student, the student must be made aware of what was wrong and why they were punished. Teachers should let students know that punishment is the inevitable result of their own behavior. All students cannot be punished for the fault of an individual or a few. It would be unjustifiable not to punish all students for speaking in class. Teachers must not use any form of punishment that violates laws and regulations and must protect students’ basic rights, such as the right to privacy, the right to education, and personal rights.

3. The principle of appropriate punishment.

Punishment must be commensurate with the mistakes made by the students and must conform to the laws of students’ physical and mental development. In fact, punishment should be a helpless choice based on the ineffectiveness of all flexible educational means. Students are in a stage of instability, both psychologically and behaviorally. It is normal for them to make small mistakes, and teachers cannot discipline students for small mistakes. Sometimes a look or an expression can achieve the purpose of education, and can effectively stop wrong behavior. Likewise, if a student makes a serious mistake, the teacher cannot just sit back and let it go. Punishment can only be effective if certain mistakes are given certain punishments.

4. Principles of Effective Punishment.

Disciplinary is a painful experience for students in every sense of the word. Therefore, punishment must be effective. In this way, this punishment is worthwhile for students and teachers. It is an inhumane act to implement it knowingly that it has no effect. When implementing discipline, the individual differences of students, such as family environment and personality, should be fully considered first. To limit it to what they themselves can afford now and can benefit from in the future. Second, it needs to seize the moment. Whether adults or children, when they know that they have made a mistake, they will have a mental preparation in their hearts to accept punishment. At this time, it is in line with psychological needs to take certain punishments and balance the guilt of making mistakes. Finally, punishment must be fair, just, open, and treated equally. Only in this way can education be effective.

C. Mastering disciplinary skills.

What Chinese education lacks is not punishment but wisdom.

Of course, it is difficult to punish students wisely. It should fully take into account factors, such as students’ character, and choose an appropriate method to achieve the desired effect. Punishing students wisely can also reflect the good intentions of teachers. For example, some students like extracurricular activities, and teachers can cancel extracurricular activities for a period of time after students do not do homework as punishment. Of course, if the student is introverted and does not like sports, such punishment is like a reward and has no effect. This requires teachers’ punishment measures to be expressed wisely and in good faith on the basis of fully understanding students.

## Teaching and Learning Optimization Algorithms

This paper adopts the teaching and learning optimization algorithm (also known as the TLBO algorithm). In algorithms, a class is a population in a search space. The number of students in a class is the number of populations, and all courses of students are the dimension of a population.


(1) $$\sigma \left(\mu \right)=\frac{1}{\sqrt{2\pi \lambda }}\exp\left(-\frac{{\left(\mu -\nu \right)}^{2}}{2\lambda^{2}}\right)$$

Among them, ν is the mean, $\lambda^{2}$ is the variance, and μ is the variable that obeys the normal distribution.

To a certain extent, it improves the overall level of the class teaching process:


(2) $$\gamma^{1}=\gamma^{0}+\psi$$

(3) $$\psi =\tau \cdot \left(\gamma^{t}-\theta '\beta \right)$$

Among them, $\gamma_{2}$ is the optimal individual in the population, that is, the teacher, θ is the teaching factor, β is the average grade of the entire class, and τ is the learning step length. It takes random numbers equally distributed between 0 and 1.


(4) $$\theta =\mathrm{round}\left(\tau +1\right)$$

Learning process:


(5) $$\gamma_{n}^{1}=\left\{\begin{array}{c} \gamma^{0}+\tau \cdot \left(\gamma^{s1}-\gamma^{0}\right)\;\;\\ \gamma^{0}+\tau \cdot \left(\gamma^{s2}-\gamma^{0}\right)\;\; \end{array}\begin{array}{c} \;\;if\;\;\sigma \left(\gamma_{s1}\right)<\sigma \left(\gamma_{s2}\right)\\ otherwise \end{array}\right\}$$

Where $\gamma_{s1}$ and $\gamma_{s2}$ are two different students drawn at random.

It initializes the population, obtains an initial population, and calculates the individual fitness value of the population.


(6) $$\gamma_{n,m}=\gamma_{m,\min}+\tau \cdot \left(\gamma_{\max}-\gamma_{\min}\right)$$

In the I-TLBO algorithm, the teaching factor is designed in an adaptive way. Teaching factor θ is modified to:


(7) $${\left(\theta \right)}_{n}={\left(\frac{\gamma_{c}}{\gamma_{d}}\right)}_{n}$$

(8) $$c=1,2,\ldots ,\varepsilon \;if\gamma_{c,n}\neq 0$$

(9) $${\left(\theta \right)}_{n}=1\;\;if\gamma_{c,n}=0$$

Among them, $\gamma_{c}$ is the student c, and $\gamma_{d}$ is the best individual in the population.

In the I-TLBO algorithm, the tutoring training is classified into the teacher teaching stage:


(10) $${\left(\gamma_{m,p}^{'}\right)}_{r}={\left(\gamma_{m,p}+\psi \_\beta_{m}\right)}_{r}+\tau {\left(\gamma_{\kappa }-\gamma_{p}\right)}_{r}\;\;if\sigma {\left(\gamma \right)}_{\kappa }>\sigma {\left(\gamma \right)}_{p}$$

(11) $${\left(\gamma_{m,p}^{'}\right)}_{r}={\left(\gamma_{m,p}+\psi \_\beta_{m}\right)}_{r}+\tau {\left(\gamma_{p}-\gamma_{\kappa }\right)}_{r}\;\;if\sigma {\left(\gamma \right)}_{\kappa }<\sigma {\left(\gamma \right)}_{p}$$

Among them, κ≠p .

Students can also learn through self-motivation to improve their grades:


(12) $${\left(\gamma_{m,p}^{'}\right)}_{r}=\gamma_{m,p,n}^{'}+\tau {\left(\gamma_{m,p}^{'}-\gamma_{m,\rho }^{'}\right)}_{r}+\tau {\left(\gamma_{t}-\omega \gamma_{m,p}^{'}\right)}_{r}\;if\sigma \left(\gamma_{p}^{'}\right)>\sigma \left(\gamma_{\rho }^{'}\right)$$

(13) $${\left(\gamma_{m,p}^{'}\right)}_{r}=\gamma_{m,p,n}^{'}+\tau {\left(\gamma_{m,\rho }^{'}-\gamma_{m,p}^{'}\right)}_{r}+\tau {\left(\gamma_{t}-\omega \gamma_{m,p}^{'}\right)}_{r}\;if\sigma \left(\gamma_{p}^{'}\right)<\sigma \left(\gamma_{\rho }^{'}\right)$$

Where ω is the exploration factor and *r* is the *r*th subpopulation.

Crossover operation:


(14) $$h_{m}=\left\{\begin{array}{cc} \gamma_{m}^{1} & if\tau <\eta \;\;or\;\;m=\tau 'A\\ \gamma_{m}^{0} & otherwise \end{array}\right\}$$

Where η is the crossover rate and *A* is the dimension of the problem being solved.

Global reverse number:


(15) $$\mu^{0}=e+f-\mu$$

Among them, μ∈(e,f) .

Global reverse point:


(16) $$\mu_{n}^{0}=e_{n}+f_{n}-\mu_{n}\;\;,n=1,2,3,\ldots ,A$$

Where $\mu_{n}\in \left(\mu_{1},\mu_{2},\ldots ,\mu_{A}\right)$ is a point in A-dimensional space.

When τ<0.5 , the improved multi-learning method is:


(17) $$\gamma_{n}^{1}=\left\{\begin{array}{cc} \gamma_{n}^{0}+\tau \cdot \left(\gamma_{n}^{0}-\gamma_{n}^{s1}\right) & \sigma \left(\gamma_{0}\right)<\sigma \left(\gamma_{s1}\right)\\ \gamma_{n}^{0}+\tau \cdot \left(\gamma_{n}^{s1}-\gamma_{n}^{0}\right) & otherwise \end{array}\right\}$$

When τ≥0.5 , the improved multi-learning method is:


(18) $$\gamma_{n}^{1}=\left\{\begin{array}{cc} \gamma_{n}^{0}+\tau \cdot \left(\gamma_{n}^{s2}-\gamma_{n}^{s1}\right) & \sigma \left(\gamma_{s2}\right)<\sigma \left(\gamma_{s1}\right)\\ \gamma_{n}^{0}+\tau \cdot \left(\gamma_{n}^{s1}-\gamma_{n}^{s2}\right) & otherwise \end{array}\right\}$$

Among them, $\gamma^{s1}$ and $\gamma^{s2}$ are two random individuals different from $\gamma^{0}$ in the population.

Population initialization:


(19) $$\gamma_{n,m}=\gamma_{m,\min}+\tau \cdot \left(\gamma_{\max}-\gamma_{\min}\right)$$

Test function:


(20) $$\sigma_{\mu \iota \nu }=\overset{Α}{\underset{\nu =1}{\sum }}\gamma_{\nu }^{2},\gamma_{\nu }\leq 100$$

Four hundred ninety-three questionnaires were randomly distributed from University X and University Y from China, and 389 valid questionnaires were obtained after screening. The actual sample distribution is shown in Table 1.

**Table 1:** Sample distribution.

Projects	Demographic variables	Effective sample size (*N* = 389)	Percentage (%)
Urban and rural	City	126	32.39
	Rural	263	67.61
Gender	Male	217	55.78
	Female	172	44.22
Grade level	Freshman year	87	22.37
	Sophomore	105	26.99
	Third year	113	29.05
	Senior year	84	21.59

**Figure 5:**
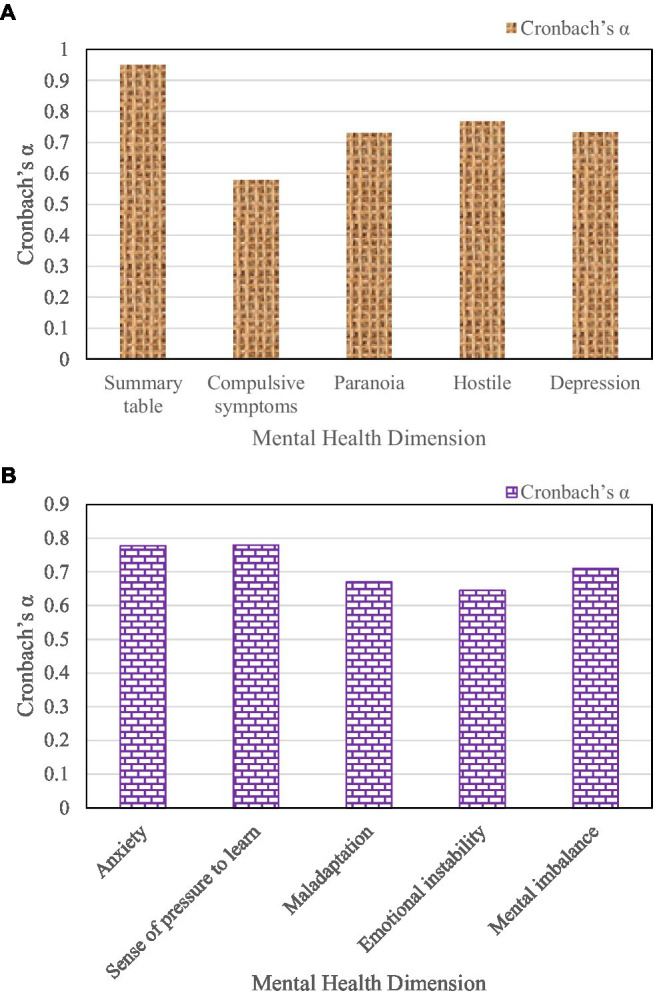
Cronbach’s coefficients for each dimension of the Student Mental Health Rating Scale.

The Cronbach’s coefficients of each dimension of the College Students’ Mental Health Rating Scale are shown in Figure 5.

As shown in Figures 5A,B the overall Cronbach’s coefficient of the scale is 0.951 after the reliability test of the College Students’ Mental Health Rating Scale. And the Cronbach’s coefficient of “anxiety” is 0.777, and the Cronbach’s coefficient of “depression” is 0.733. And the Cronbach’s coefficients of each dimension are mostly above 0.7. This indicates that the College Student Mental Health Rating Scale has high internal consistency reliability in this measurement.

**Figure 6:**
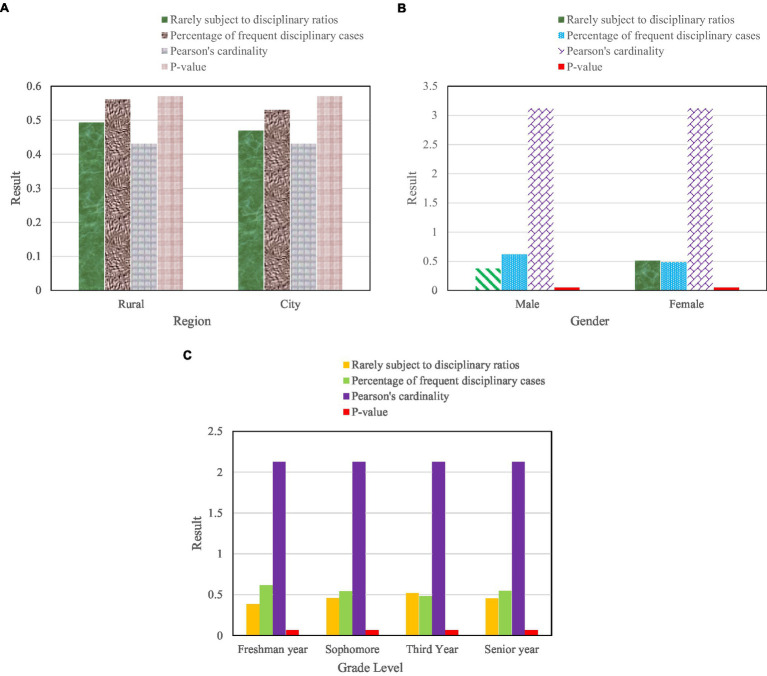
Cardinality test results for teacher discipline on the urban–rural and gender dimensions. [**(A)** Test results of geographical dimension, **(B)** Test Results for Gender Dimension, and **(C)** Test results of grade dimension].

In order to find out the differences between urban and rural areas, genders, and grades of teacher punishment, the chi-square test was used to compare the differences between urban and rural areas and gender and grades. The specific results are shown in Figure 6.

It can be seen from Figure 6A that there is no significant difference between urban and rural areas in teacher discipline. That is to say, there is no significant difference between urban and rural college students in the situation of being disciplined by teachers. Figure 6B shows that the chi-square test results show that teacher discipline differs significantly in the dimension of gender. That is, boys are more likely to be punished than girls. It can be seen from Figure 6C that there is no significant difference in teacher discipline in the dimension of grade.

The differences in the coping styles of college students in the dimensions of urban and rural areas, gender and grade are shown in Tables 2–4.

**Table 2:** Analysis of the differences in coping styles between university students on the urban and rural dimensions.

Dimensions of the easy response approach	Urban and rural		*t*-value
	Cities (*N* = 126)	Rural (*N* = 263)	
Active response	1.2349 ± 0.41862	1.3479 ± 0.71232	0.759
Negative response	1.1748 ± 0.34768	1.4327 ± 0.41865	0.330

**Table 3:** Analysis of the differences in coping styles of university students on the gender dimension.

Dimensions of the easy response approach	Gender		*t*-value
	Male (*N* = 217)	Female (*N* = 172)	
Active response	1.4189 ± 0.34819	1.7189 ± 0.51203	−3.749
Negative response	1.1743 ± 0.81432	1.0175 ± 0.41861	1.207

**Table 4:** Analysis of differences in college students’ coping styles along the grade dimension.

Dimensions of the easy response approach	Gender				*t*-value
	Freshman year (*N* = 87)	Sophomore (*N* = 105)	Third year (*N* = 113)	Senior year (*N* = 84)	
Active response	1.3149 ± 0.50715	1.4823 ± 0.49152	1.8147 ± 0.60923	1.4620 ± 0.30718	2.945
Negative response	1.3018 ± 0.39168	1.2043 ± 0.71529	1.0713 ± 0.41934	1.5017 ± 0.40716	3.149

It can be seen from Table 2 that the subscale of positive coping style for urban college students scored 1.2349. There is no significant difference in the choice of coping styles among college students in different regions.

It can be seen from Table 3 that the positive coping style subscale score of male college students is 1.4189, and the positive coping style subscale female college students score is 1.7189. The negative coping style subscale male college students scored 1.1743, and the negative coping style subscale female college students scored 1.0175.

The positive coping style subscale score of freshman students was 1.3149, and the negative coping style subscale freshman student score was 1.3018. It can be seen from Table 4 that there are significant grade differences in the scores of the two coping style subscales.

**Figure 7:**
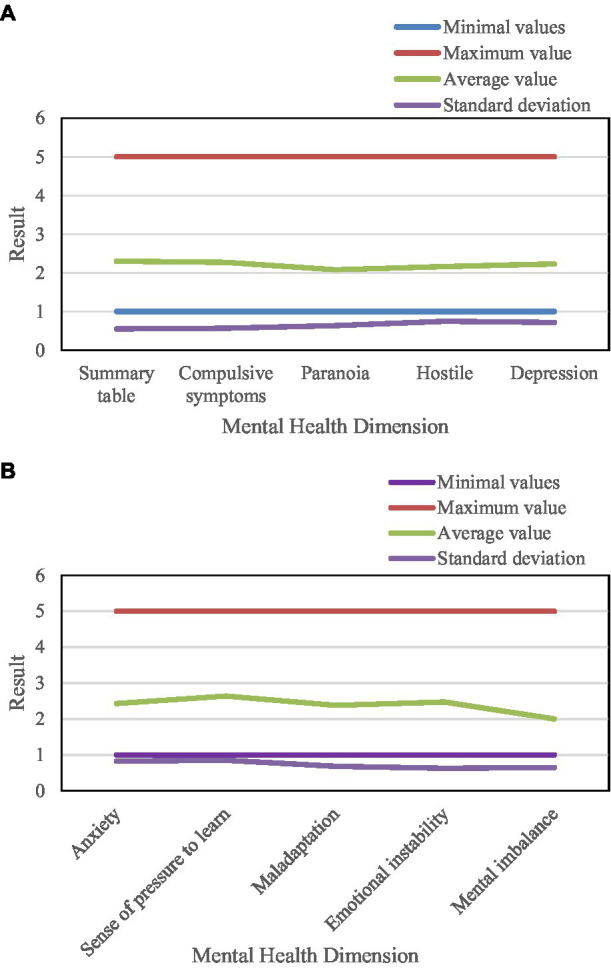
Overall mental health of university students.

The overall situation of college students’ mental health is shown in Figure 7.

Figure 7 shows a score of 2.00 for “mental imbalance” and a score of 2.47 for “emotionally unstable.” “Anxiety” scored 2.43 and “Depression” scored 2.23. In addition, college students scored more than two points in all dimensions of mental health. Among the subscale scores, the “study pressure” score is relatively high. This means that these high school students generally have a high sense of learning pressure.

Students’ understanding of the role of punishment is shown in Table 5.

**Table 5:** Questionnaire on perceptions of the role of discipline (students).

Options	Select number of people		Proportion (%)
	City	Rural	
Effective	89	194	72.75
No effect	12	23	9.00
I cannot say	25	46	18.25

Table 5 shows that 72.75% of students think that punishment is effective. The proportion of students who think that punishment has no effect is 9%, and the other 18.25% of students cannot explain the effect of punishment. Generally speaking, most students still think that punishment is effective, so punishment still has its actual meaning.

The attitudes of students toward punishment are shown in Table 6.

**Table 6:** Students’ attitudes toward discipline.

Options	Select number of people		Proportion (%)
	City	Rural	
Should	103	214	81.49
Shouldn’t	12	31	11.05
Does not matter	11	18	7.46

Table 6 shows that 81.49% of the students agree with the method of discipline, and only 11.05% of the students do not agree with the method of discipline. Nowadays, most college students still have a rational understanding of punishment, and they will not blindly regard “punishment” as “corporal punishment.”

**Figure 8:**
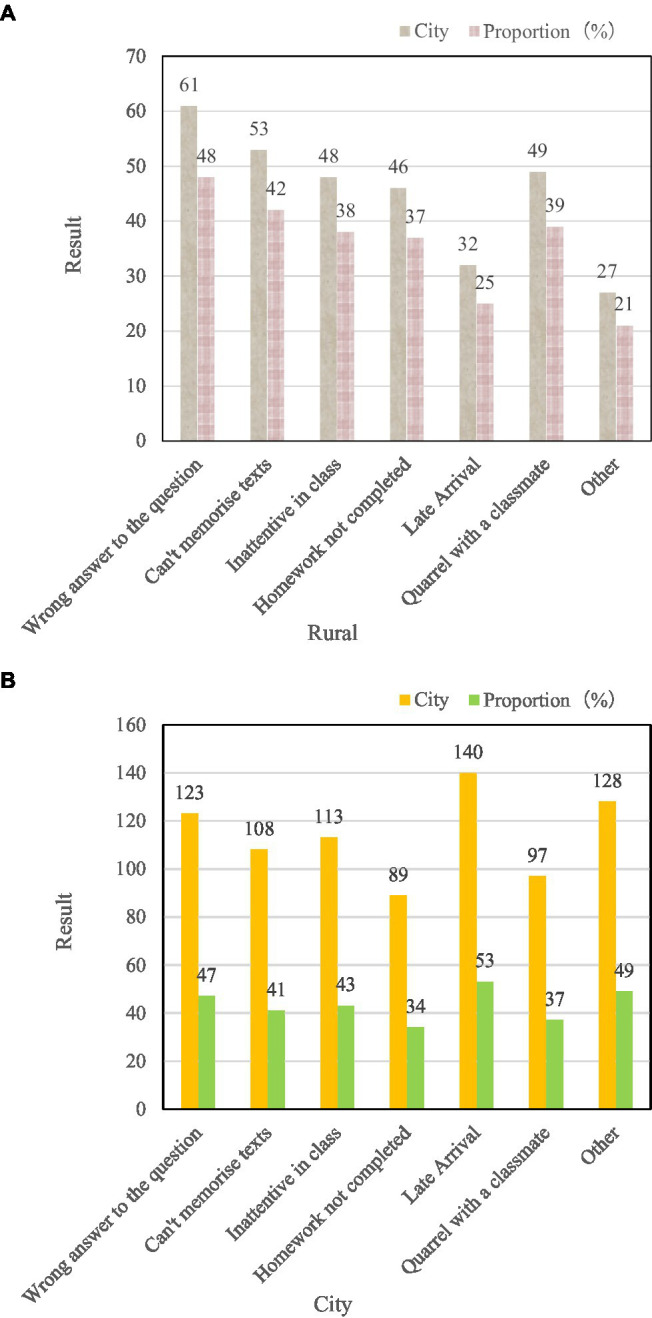
Survey of students’ reasons for receiving discipline in different districts.

Figure 8 shows the investigation of the reasons why students in different regions receive punishment.

Figure 8 shows that the reasons for students receiving discipline can be summarized as: intellectual problems and violation of basic code of conduct. Among them, urban students have the highest probability of being punished for being late, reaching 53%. The probability of being disciplined for not completing homework was the lowest at 34%. Rural students had the lowest probability of being disciplined for being late, at 25%, and had the highest probability of being disciplined for answering questions incorrectly, at 48%.

## Discussion

Improving the system and procedure of teacher disciplinary action through legislation is the necessary prerequisite for the rational exercise of the teacher’s disciplinary power. The value of education is the meaning of the actual educational phenomenon to the individual. It generally exists in human education and social practice and is closely related to human life. In terms of the relationship between truth and value, the understanding of educational value is formed in education, and it is the test standard for practical activities in human society. Whether it is from the perspective of the history of education development, the purpose of education, and the education system, the rational value of its existence no longer needs to be verified. However, improving the system and procedure of teacher punishment through legislation still needs to be continuously promoted. The use of discipline aims to make students realize their own mistakes and grow in the teacher’s discipline method, which is undoubtedly beneficial to students. However, in terms of the impact on students’ health, although the original intention is good, it depends on how students view disciplinary behaviors individually. Maybe some students think that they should be disciplined, so not only is there no harm in psychology, but it may be helpful. However, if the students mistakenly think that the teacher’s disciplinary behavior is “corporal punishment,” it will still have a negative impact on the students.

## Conclusion

The original purpose of discipline is to help students grow from mistakes. But in today’s environment, it seems that teachers really “seeking truth from facts” punishment has become a luxury. Teachers either do not dare to punish students for fear that students or the intensity of discipline exceeds the “six principles” of discipline. But for teachers, violating the laws of students’ physical and mental development, punishing or disguised corporal punishment of students or disregarding their educational responsibilities. It is morally inadmissible to ignore students and ignore what should be done. We hope that teachers can fulfill their duties, complete their educational tasks, set a good example for students in moral learning, and live up to the high expectations of students, parents, and the society. This research mainly uses the survey method to discuss the use of teachers’ disciplinary power in students’ teaching. What the paper presents is only the author’s current understanding of the issue. Due to the limitations of research methods and research conditions, no empirical research on this issue has been carried out. Many problems in real teaching activities fail to enter the field of research. In this sense, it can be said that the completion of this thesis is only a summary of the research, far from the final result of the research. In the future research process, the author will add the results of the empirical research and expect to draw more mature conclusions.

## Data Availability Statement

The original contributions presented in the study are included in the article/supplementary material; further inquiries can be directed to the corresponding author.

## Author Contributions

The author confirms being the sole contributor of this work and has approved it for publication.

## Conflict of Interest

The author declares that the research was conducted in the absence of any commercial or financial relationships that could be construed as a potential conflict of interest.

## Author’s Note

ZA was born in Tianshui, Gansu, China, in 1989. He received the doctor of law at Zhejiang University, China. Now, he works in School of Law and Intellectual Property, Foshan University. His research interests include Jurisprudence, Judicature, Law culture, Law of education.

E-mail: azhmfo@fosu.edu.cn.

## Publisher’s Note

All claims expressed in this article are solely those of the authors and do not necessarily represent those of their affiliated organizations, or those of the publisher, the editors and the reviewers. Any product that may be evaluated in this article, or claim that may be made by its manufacturer, is not guaranteed or endorsed by the publisher.
